# Approaching motor and language deficits in autism from below: a biolinguistic perspective

**DOI:** 10.3389/fnint.2015.00025

**Published:** 2015-03-30

**Authors:** Antonio Benítez-Burraco, Cedric Boeckx

**Affiliations:** ^1^Department of Spanish Philology and its Didactics, University of HuelvaHuelva, Spain; ^2^Catalan Institute for Advanced Studies and Research (ICREA)Barcelona, Spain; ^3^Department of Linguistics, Universitat de BarcelonaBarcelona, Spain

**Keywords:** ASD, motor deficits, language, speech, candidate genes, language-ready brain, externalization, vocal learning

In their paper “Oral motor deficits in speech-impaired children with autism” (*Front. Integr. Neurosci*. 7:47). Belmonte et al. (2013) argue that expressive language deficits in a subgroup of people with autism result from the impairment of the oromotor function. As a matter of fact, the paper appeared in a Frontiers Research Topic that brings movement to the forefront of autism research. Ultimately, this collection of papers aims to support the view that movement and cognition should be considered jointly if we want to properly diagnose and treat this condition, and that motor abnormalities may constitute a significant and robust endophenotype for autism spectrum disorders (ASD) (see Esposito and Paşca, 2013). At the same time, advances in genome-wide technology have yielded an increasing amount of genes related to autism, which point to specific mechanisms and pathways underlying its associated deficits (Jeremy Willsey and State, 2014). However, as pointed out by Jeste and Geschwind (2014) robust endophenotypes of the disorder based on these findings are still forthcoming and the gap between the pathophysiology of autism and genes still remains open. The goal of this commentary is to contribute to bridge this gap between genes and ASD, focusing specifically on motor dysfunction and language deficits in people with autism. In doing so, we will adduce considerations from the fields of the biology of language and of language evolution (aka *Biolinguistics*). We hope that steps of the sort we take will eventually help us better understand the comorbidity, heterogeneity, and variability of ASD, but also the biological underpinnings of the human faculty of language.

In our recent research (Boeckx and Benítez-Burraco, 2014a,b; Benítez-Burraco and Boeckx, submitted) we have put forth three set of genes that we think are important for the emergence of the two basic components of our species-specific capacity for acquiring and using a language, which we have labeled our “language-ready brain”: our specific mode of cognition (namely, the ability to form cross-modular concepts), and our specific way of externalizing thoughts, usually in the form of strings of sounds. In the linguistic literature these two separated abilities are usually referred to as the syntax-semantics interface and the morphophonological component, respectively.

The first set of genes is centered around *RUNX2*, a gene involved in skull and brain development. According to our findings, the evolutionary modification of this network may account for our more globular head shape (compared to Neanderthals) and for the concomitant rewiring of different connections between cortical and sub-cortical (specifically, thalamic) structures, which provide the scaffolding for our species-specific mode of cognition (see Boeckx and Benítez-Burraco, 2014a for details). The second set is centered on the *ROBO1* and *FOXP2* interactomes, two genes that have been repeatedly associated to speech disorders, and which emerge from the literature as prominent molecular signatures of vocal learning and motor control (see Boeckx and Benítez-Burraco, 2014b for details). The third set is clustered around *AUTS2* and it provides additional robust links between the two main networks we have uncovered (see Benítez-Burraco and Boeckx, submitted for details). Many components of these three sets show fixed changes in modern humans compared to Neanderthals/Denisovans, which affect their regulatory regions, their coding regions, or both. Overall, we think that most of the genetic changes that brought about our language-readiness did not occur in a mosaic fashion, but rather revolved around changes in skull/brain morphology and neural connectivity that resulted in a more globular brain.

It is with this background in mind that we want to approach Belmonte et al.'s (2013) paper and ultimately, the motor perspective on language dysfunction in autism. What we have found is that many candidates for ASD belong to the three gene sets we believe are important for language development in the individual and in the species. For example, as part of our set centered on *RUNX2* one finds *CTNNB1* (O'Roak et al., 2012), *HRAS* (Comings et al., 1996), *DLX1* (Liu et al., 2009), *DLX5* (Nakashima et al., 2010), *PTEN* (Naqvi et al., 2000; Butler et al., 2005), and *SMURF1* (De Rubeis et al., 2014), all potential ASD candidates. Similarly, one finds several candidates for ASD among the genes comprising the second set, centered on the *FOXP2*/*ROBO1* interactomes, including *ROBO2* (Suda et al., 2011), *FOXP1* (Hamdan et al., 2010), *POU3F2* (Lin et al., 2011), and *CNTNAP2* (Alarcón et al., 2008). Finally, *AUTS2* (Oksenberg and Ahituv, 2013) is a robust candidate for ASD, and this is also the case with other genes that are functionally linked to it, like *TBR1* (Deriziotis et al., 2014), *FEZF2* (Wang et al., 2009), *PAX6* (Maekawa et al., 2009) and *SATB2* (Kwan, 2013).

When we move from genes to cellular function, we find that these three set of genes, and specifically the ones we just highlighted, regulate processes that are central in the pathophysiology of autism. Concerning the set centered on *RUNX2*, it is primarily related to skull morphogenesis, thalamic development and the specification, migration and interconnection of GABAergic neurons within the forebrain. Interestingly, autism sometimes entails deviations in head shape, resulting from deviations in brain growth and connectivity (Cheung et al., 2011). More importantly, aberrant development of GABAergic interneurons has been linked to ASD (Di Cristo, 2007). Lastly, autism usually involves the impairment of thalamocortical connectivity (Nair et al., 2013). The thalamus acts as an efficient mode of cortico-cortical connections that we deem necessary for cross-modularity and for going beyond stereotypical behaviors (see Boeckx and Benítez-Burraco, 2014a for details). Overall, given the role of this set of genes in language development and evolution, the fact that we find among them several autism-candidate genes may help explain the distinctive mode of cognition observed in people with autism, but also their problems with (receptive) language (e.g., Tager-Flusberg et al., 2005).

The thalamus also functions as a bridge between several cortical and subcortical components important for the externalization of language, as part of the so-called anterior pathway of vocal learning, which involves the basal ganglia (especially, the striatum) and the fronto-temporal network connecting Wernicke's and Broca's areas (Hickok and Poeppel, 2007; Miller and Buschman, 2007; Petkov and Jarvis, 2012). All these brain areas and neuronal networks show anomalies in ASD (e.g., Murphy et al., 2014). Specifically, tracts between the thalamus and the motor cortex are compromised in ASD, suggesting that the former region may play a role in motor abnormalities reported in this condition (Nair et al., 2013). Overall, the fact that we find several autism-candidate genes among the genes that are related to the externalization of language may explain why speech problems are frequently observed in people with autism. As noted by Wang et al. (2015) this may be due to the involvement of the ROBO proteins and their ligand SLITs, which play a central role in the organization of the specialized forebrain circuits that control vocal learning, specifically, the forebrain part that connects to brainstem vocal motor neurons (Wang et al., 2015), and which points to convergent molecular changes in all vocal learners (Pfenning et al., 2014). The involvement of FOXP2 and its partners makes this link even more robust and more interesting for us, since they play a key role in the development and function of cortico-thalamic-striatal circuits contributing to efficient motor planning and execution (Fisher and Scharff, 2009; Schreiweis et al., 2014). Interestingly, some of these proteins function in conjunction with ROBOs and SLITs (e.g., CNTNAP2) (Banerjee et al., 2010). Finally, the constellation of genes around *AUTS2* is important for the establishment of intra- and interhemispheric connectivity and in particular, of an optimal balance between excitation and inhibition, which seems to be also impaired in autism (Just et al., 2004; Yizhar et al., 2011; Zikopoulos and Barbas, 2013).

Returning to our initial concern of the heterogeneity, variability, and specifically, comorbidity between motor and cognitive disorders observed in ASD, this frequent outcome of research and of clinical practice may be explained by the fact that the three set of genes we highlight here, involved in different aspects of cognition and motor behavior, are functionally interconnected. For example, as we mentioned earlier, some people suffering from autism bear a defective copy of *TBR1*. Specifically, disruptions of *TBR1* give rise to severe speech and language deficits, autistic problems, and moderate to severe intellectual disability (Palumbo et al., 2014). This complex, comorbid phenotype may be partially explained by the molecular interactions TBR1 is involved in. To begin with, *TBR1* interacts with *FOXP2* (Deriziotis et al., 2014), which is relevant for the externalization of language. Moreover, *TBR1* seems to act as a master regulator controlling several other ASD-candidate genes (Chuang et al., 2015), including AUTS2 (Bedogni et al., 2010). Among *Auts2* regulators we find both *Runx2* and *Foxp2* (Oksenberg et al., 2014), which are central pieces of our networks. At the brain level *TBR1* contributes to the establishment of neocortical connectivity with the thalamus (McKenna et al., 2011), but also of interhemispheric connections involving callosal axons (Hevner et al., 2001). Importantly, the integrity of the corpus callosum is frequently reported to be affected in people suffering from ASD (Kumar et al., 2010; Shukla et al., 2010). Similarly, a decrease in interhemispheric connections seems to be a hallmark of this condition (Shukla et al., 2010; Ingalhalikar et al., 2011). Lastly, in mice *Tbr1* haploinsufficiency also results in defective axonal projections of amygdalar neurons, which give rise to a deficit in ultrasonic vocalization, social interaction, and associative memory and cognitive flexibility (Huang et al., 2014).

It is also of interest that many of the genes we have focused on have changed after our split from Neanderthals and Denisovans (see Boeckx and Benítez-Burraco, 2014a,b; Benítez-Burraco and Boeckx, submitted for details). It has been hypothesized that recently evolved neuronal networks are more sensitive to damage because they are endowed with less robust compensatory mechanisms (Toro et al., 2010). As a consequence, perturbations affecting them (e.g., mutations in specific genes) are expected to impair their development more easily and more frequently. This could account for the high prevalence of autism within modern human populations (according to Gibson, 2009 disorders like ASD are de-canalized conditions, resulting from the uncovering of cryptic genetic variation as a consequence of genomic, environmental, or even cultural perturbations).

In sum, we regard the motor approach to language dysfunction in autism of outstanding interest for autism research, but also for our understanding of the biological nature of the human faculty for language. We believe that the genetic aspects highlighted here may contribute to gain a better understanding of the way in which cognitive and motor behaviors affect each other in people with ASD.

## Conflict of interest statement

The authors declare that the research was conducted in the absence of any commercial or financial relationships that could be construed as a potential conflict of interest.
